# Should Consumptives Be Segregated?

**Published:** 1908-02-08

**Authors:** 


### PUBLIC HEALTH AND HYGIENE.

### SHOULD CONSUMPTIVES BE SEGREGATED?

In the annual report of the Public Health Committee of the London County Council Sir Shirley Murphy, the medical officer of health of the county, discusses, *inter alia*, the important question of the segregation of persons suffering from tuberculosis.

The answer to the question will obviously in large measure be determined ultimately by the ætiological views which are held in regard to this disease. Sir Shirley Murphy therefore very properly addresses himself to the problem of the ætiology of tuberculosis. "It is impossible not to recognise a trend of opinion in the direction of regarding the phthisical person as a public danger. It becomes, therefore, very necessary," he says, "to examine the grounds upon which a demand is made which, if fully acted upon, would have the effect of depriving many thousands of persons in London of their liberty."

It has been urged as evidence of the danger arising from contact with phthisical persons and their immediate environment that the houses and people with whom they dwell are disproportionately associated with the disease. Dealing with this association Sir Shirley Murphy shows that statistically it is little more than is accounted for by arithmetical probability.

"Further," he adds, "it must be recollected that if malnutrition, overcrowding, ill-ventilation, and similar concomitants of poverty play an important part in the causation of phthisis it might be expected that, quite apart from the question of infectivity of the disease, more deaths would occur in houses occupied by poor persons than in houses occupied by persons who are well-to-do, and that if it had been possible to make allowance for this the proportion of deaths occurring in houses previously invaded would have been still less conspicuous.

A similar conclusion is arrived at in regard to the association of the sufferer with other persons affected by the disease. The statistical investigations of Dr. Longstaffe and Professor Karl Pearson referred to in the report have failed to show that the husband or wife of a tuberculous mate becomes infected with greater frequency than mere arithmetical probability would lead one to anticipate. What does come out as a result of Professor Karl Pearson's inquiry, as Sir Shirley Murphy points out, is the influence of heredity upon the liability to attack.

"I feel fairly certain," says Professor Pearson, "that for the artisan class the inheritance factor is far more important than the infection factor, because in a very large proportion of cases it does not lie in the power of the individual to maintain in the stress of urban life a wholly safe environment. I am inclined to think that . . . the constitution or diathesis means almost everything for the individual whose life cannot be spent in self-protection."

Since Koch's discovery of the tubercle bacillus the hyper-susceptibility to tubercular infection which is a well-marked and unfortunate hereditary trait in so many families has been largely lost sight of in the advancement of a crusade directed solely against the external conditions favouring the disease. These views have culminated in the demand noted by Sir Shirley Murphy for segregation of phthisical persons, and have led him to restate the broad pathological facts regarding tuberculosis, on which alone sound administrative action can be based. "The acceptance of the view that under the conditions which exist in urban populations the susceptibility of the individual and not the exposure to known cases of phthisis governs the probability of attack does not itself negative the theory of infectivity of tubercular phthisis," he goes on to tell us.

That bovine tuberculosis may play an essential part in the production of human tuberculosis is definitely shown as a result of experiments recently conducted at the instance of the Royal Commission on Tuberculosis. Some of the conclusions arrived at by the Commission are quoted by Sir Shirley Murphy as follows

"There can be no doubt but that, in a certain number of cases, the tuberculosis occurring in the human subject, especially in children, is the direct result of the introduction into the human body of the bacillus of bovine tuberculosis, and there also can be no doubt that in the majority at least of these cases the bacillus is introduced through cow's milk. . . . A very considerable amount of disease and loss of life, especially among the young, must be attributed to the consumption of cow's milk containing tubercle bacilli. The presence of tubercle bacilli in cow's milk can be detected, though with some difficulty, if the proper means be adopted, and such milk ought never to be used as food. There is far less difficulty in recognising clinically that a cow is distinctly suffering from tuberculosis, in which case she may be yielding tuberculous milk. The milk coming from such a cow ought not to form part of human food, and, indeed, ought not to be used as food at all. Our results point to the necessity of measures, more stringent than those at present enforced, being taken to prevent the sale or the consumption of such milk."

These conclusions indicate clearly a line of attack in the measures to be taken to limit the spread of tubercular infection, and there can be no doubt that the freeing of our milk and meat supplies from the organisms of tubercle would stop an important channel of conveyance of infective material. Were this accomplished, however, the hope would not be justified that those with a high degree of susceptibility to tuberculosis would be freed in any appreciable measure from risk of contracting infection.

The hereditary element in the causation of the disease would remain as it is, the dominant factor in urban populations. The saving from tuberculosis would be effected chiefly among those in whom susceptibility was not a permanent physical character of their tissues, but rather an acquired and probably transient loss of natural immunity due to innutrition, a bad hygienic environment, or other predisposing condition of tubercular infection.

